# Biochemical analysis of a TrkB receptor mutation that causes a developmental epileptic encephalopathy

**DOI:** 10.1016/j.jbc.2026.111152

**Published:** 2026-01-10

**Authors:** Yang Zhong Huang, Cormic McNamara, James O. McNamara

**Affiliations:** 1 Department of Neurobiology, Duke University School of Medicine, Durham, North Carolina, USA; 2 Department of Neurology, Duke University School of Medicine, Durham, North Carolina, USA; 3 Departments of Pharmacology and Cancer Biology, Duke University School of Medicine, Durham, North Carolina, USA

**Keywords:** BDNF, disulfide, epilepsy, phosphotyrosine signaling, protein degradation, receptor tyrosine kinase, TrkB

## Abstract

TrkB, a receptor tyrosine kinase encoded by gene *NTRK2*, is essential for diverse biological processes in both the developing and mature mammalian nervous systems. Whole exome sequencing of children with developmental epileptic encephalopathy revealed an intriguing syndrome caused by a rare *de novo* recurrent variant of TrkB, namely Y434C. Investigating the biochemical properties of the Y434C mutant protein is an important initial step toward understanding how this mutation causes this devastating disease. This led us to establish and characterize multiple cell lines stably expressing mouse WT or Y434C TrkB. In comparison to WT, Y434C mutant cell lines expressed low to undetectable levels of mature form (145 kDa) of TrkB protein and varying levels of mutant forms migrating at sizes ranging from 40 to 110 kDa. Y434C mutant cell lines exhibited striking impairments of brain-derived neurotrophic factor–mediated activation of TrkB signaling. Reducing agents reduced high molecular weight forms of Y434C protein multimers detected in protein gel electrophoresis, consistent with disulfide bond formation between the Y434C mutant proteins. We propose that conversion of tyrosine to cysteine at amino acid 434 results in a novel intermolecular disulfide bond between Y434C mutant proteins, thereby modifying their structure and enhancing their proteolytic digestion. The ensuing reductions of the mature form of TrkB likely underlie impaired TrkB signaling. The proteolytic fragments of TrkB may themselves have deleterious consequences, which contribute to the phenotypic manifestations of the Y434C TrkB mutation.

TrkB, a receptor tyrosine kinase encoded by the *NTRK2* gene, is essential for the development of the mammalian central and peripheral nervous systems (1). It serves as the receptor for its canonical ligand, brain-derived neurotrophic factor (BDNF) and is expressed in virtually every neuron as well as a diversity of glia in the brain (2, 3, 4). BDNF–TrkB signaling underlies diverse functions in the mature nervous system, including neuronal survival as well as synaptic structure, function, and plasticity (5). Impaired BDNF–TrkB signaling has been implicated in diverse disorders of the nervous system, including neuropsychiatric disorders such as depression and epilepsy, as well as neurodegenerative diseases, including Alzheimer’s and Huntington’s diseases (6, 7).

Analyses of rare genetic variants linked to disease provide an opportunity to understand gene function in health and disease. Whole exome sequencing of children with developmental epileptic encephalopathy revealed an intriguing syndrome caused by one such rare recurrent variant, (c.1301A> G (p.Tyr434Cys) (Y434C) of *NTRK2* (8). The syndrome is manifest by epileptic seizures emerging at approximately 6 months of age, associated with profound intellectual and motor disability, autistic behavior, and often impairments of vision. Epileptic seizures are usually unresponsive to antiseizure medications and persist throughout life. Typically arising *de novo*, the presence of this variant in 19 unrelated individuals with this distinctive syndrome from multiple locations worldwide implicates a causal role of the Y434C variant (9).

Understanding how this genotype leads to this phenotype promises to shed light on the function of TrkB in health and disease. An initial step is to understand how the Y434C mutation modifies the biochemical properties of TrkB. Analyses of TrkB Y434C mutation by transient expression in heterologous cells revealed evidence for ligand-independent activation of TrkB and increased levels of phosphorylation of Erk1/2, a downstream signaling pathway (10). The biochemical findings together with the medically refractory epilepsy led these authors (9, 10) to propose that Y434C causes this syndrome by enhancing TrkB signaling.

In order to characterize the biochemical properties of TrkB Y434C receptors, we adopted a different strategy whereby we established and characterized multiple cell lines stably expressing either WT or Y434C mutant TrkB. The low and constant expression levels typically provided by stably transfected cells reduce the likelihood of distorting cellular signaling pathways that might result from high levels of TrkB overexpression. Analyses of these cell lines lead us to propose that this mutation causes an intermolecular disulfide bond within the transmembrane domain, thereby modifying the structures of transmembrane helices and cytoplasmic domains and facilitating their proteolytic degradation. This results in reductions of mature form TrkB, reduced activation of TrkB signaling, and increases of proteolytic fragments of TrkB, thereby contributing to the phenotypic manifestations of this unique syndrome.

## Results

**Figure 1:**
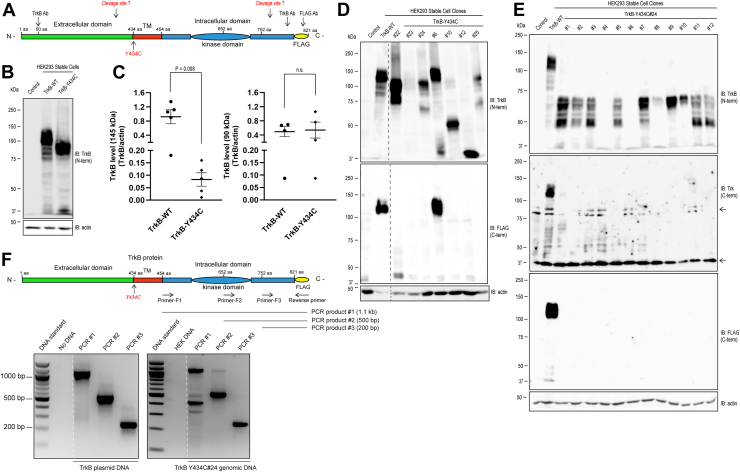
**Analysis of TrkB Y434C stably expressed in heterologous cells reveals abnormalities of protein expression and migration pattern on SDS-PAGE.** Western blot analysis of TrkB protein in *pooled* and *individual* cell lines stably expressing WT and Y434C mutant. *A*, schematic of the TrkB protein domain structure, location of the Y434C mutation, and the protein regions recognized by the antibodies for TrkB. *B*, SDS-PAGE of *pooled* WT and Y434C cell lines reveals striking differences in migrating patterns. Cell lysates were prepared from the *pooled* stable cells expressing WT or Y434C mutant, and proteins were resolved on 8% SDS-PAGE. Western blots were probed with TrkB antibody recognizing the ectodomain of TrkB. Protein loading and transfer were monitored by probing the same blots with β-actin antibody. WT protein was robustly expressed and migrated mainly at approximately 145 kDa (mature form) and at 90 kDa (immature form) to a lesser extent. In contrast, the Y434C protein migrated mainly at approximately 90 kDa. *C*, quantification of 145 kDa (*left*) and 90 kDa (*right*) of TrkB protein levels from pooled WT or Y434C expressing cells from five independent experiments. The statistical analysis was performed by Student’s *t* test, unpaired two tailed. *D*, SDS-PAGE of representative subsets of individual WT and Y434C cell lines reveals striking heterogeneity of expression level and migrating pattern of mutant protein. Aliquots of lysates of selected individual cell lines were subjected to Western blot analyses using antibodies targeting either the N-terminal ectodomain (*top blot*) or the C-terminal (targeting the FLAG epitope on the C terminus) (*bottom blot*). The antibody targeting the N-terminal ectodomain revealed a prominent 145 kDa band in WT and in one Y434C mutant cell line (#6). In the remaining Y434C mutant cell lines, striking heterogeneity was evident in both expression levels and size (ranging from 40 to 100 kDa). With respect to the C-terminal, robust immunoreactivity was evident at 145 kDa in WT and one Y434C mutant stable cell line (#6). By contrast, there was no robust immunoreactivity in the remaining Y434C cell lines. *E*, twelve subclones from TrkB Y434C #24 stable cell line were further selected and analyzed by Western blot analysis using TrkB antibodies. The TrkB N-terminal antibody detected multiple immunoreactivities migrating at approximately 40 to 60 kDa (*top panel*). The level of immunoreactivity varied among 12 subclones. In contrast, C-terminal antibodies, including Trk and FLAG antibodies (*middle and bottom panels*, respectively), failed to reveal a detectable level of immunoreactivity in these Y434C cell clones. *Arrows* in the *right margin* of the *middle panel* denote the presence of bands detected in nontransfected human embryonic kidney cells. β-actin blots in *D* and *E* were developed from separate gels. *F*, PCR detection of TrkB complementary DNA in Y434C mutant cells. *Top*, design of PCR strategy and primers amplifying TrkB DNA fragments corresponding to the intracellular domain of TrkB protein. *Bottom*, PCR amplification reveals the presence of TrkB DNA fragments from plasmid DNA (*left*) and genomic DNA isolated from Y434C #24 cell line. The *dashed lines* in *D* and *F* denote the positions at which irrelevant lanes were removed from the gel images.

**Figure 2:**
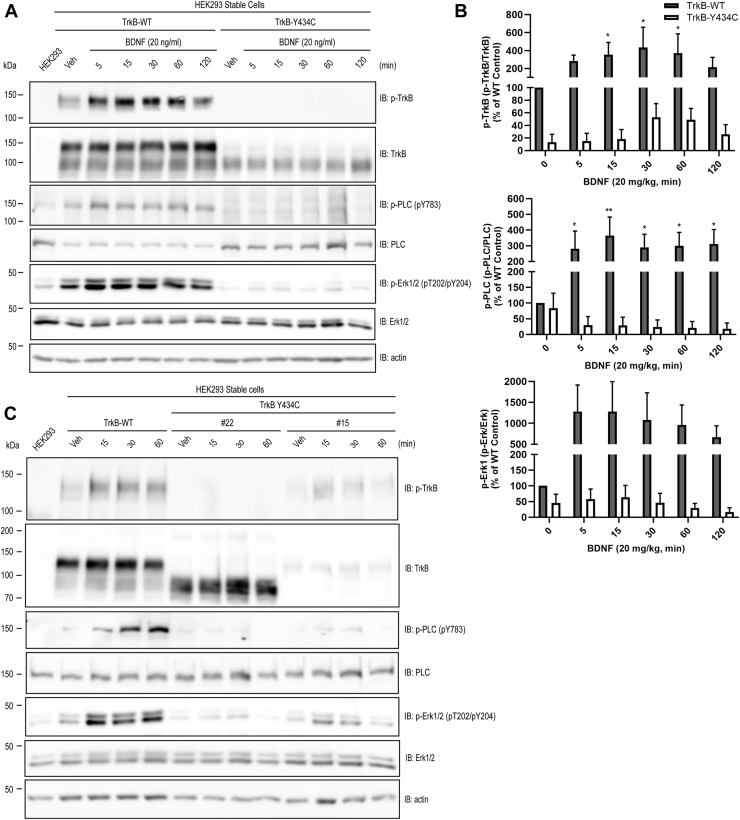
**Impaired BDNF-mediated activation of TrkB signaling in TrkB Y434C mutant cells.** Pooled (*A* and *B*) or individual (*C*) TrkB WT or Y434C stable cells were incubated with vehicle or BDNF (20 ng/ml) for the indicated periods. Cell lysates were subjected to Western blot analysis of TrkB signaling using antibodies targeting phosphorylated residues of TrkB, PLCγ1, and Erk1/2. *A*, addition of BDNF to the pooled WT cells activated TrkB signaling, evident in increased phosphorylation of TrkB and its downstream effectors PLCγ1 and Erk1/2; in contrast, BDNF-induced TrkB signaling was markedly impaired in Y434C mutant cells. Note that the Y434C mutant was mainly expressed as 90 kDa, in addition to a low level of 145 kDa protein. *B*, quantification of BDNF-induced TrkB signaling in WT and Y434C *pooled* stable cells from four independent experiments. The statistical analysis was performed by two-way ANOVA and post hoc Bonferroni’s multiple comparisons, ∗*p* < 0.05; ∗∗*p* < 0.01. *C*, responsiveness to BDNF in two representative Y434C individual mutant cell lines. WT and two representative Y434C mutant cell lines (#22 and #15) were selected to examine the efficacy of BDNF in Y434C mutants. Compared with WT cells, BDNF stimulation resulted in a modest *increase* in phosphorylation of TrkB, PLCγ1, and Erk1/2 in line #15 but not in line #22. BDNF, brain-derived neurotrophic factor; PLCγ1, phospholipase Cγ1.

To characterize the biochemical properties of a *de novo* recurrent mutation of *NTRK2* (c.1301A>G [p.Tyr434Cys]) that resides within the transmembrane domain (Fig. 1*A*), we introduced the mutation into mouse TrkB complementary DNA (cDNA), incorporated an epitope (FLAG) tag on the C terminus (Fig. 1*A*), and transfected either WT or Y434C mammalian expression plasmids into human embryonic kidney 293 (HEK293) cells. Six WT and 25 Y434C individual cell lines were established. Pools of the WT and of the Y434C mutant, including the 25 cell lines, were also established. Initial experiments characterized the expression and migration patterns of TrkB in lysates of the *pooled* cell lines resolved on SDS-PAGE. WT protein migrated predominantly at 145 kDa (the mature form) with lesser amounts at 90 to 100 kDa (the immature form) (Fig. 1, *B* and *C*), findings consistent with prior work (11). In stark contrast, there was virtually no detectable 145 kDa band in lysates of pools of Y434C cell lines. Instead, TrkB Y434C migrated predominantly at approximately 90 kDa (Fig. 1, *B* and *C*). To gain further insight into the expression pattern of the Y434C mutant protein, we conducted Western blot analyses of 25 *individual* cell lines expressing Y434C. Antibodies targeting the N-terminal and C-terminal portions of the TrkB protein were employed for immunoblotting analysis. The results revealed a highly heterogeneous pattern of Y434C protein in a representative subset of individual cell lines with respect to both expression level and migration pattern on SDS-PAGE as detected by the N-terminal TrkB antibody (*top panel* of Fig. 1*D*). As expected, WT stable cells expressed TrkB protein predominantly at 145 kDa (Fig. 1*D*). Similar to that of the lysates isolated from pooled Y434C stable cells, the majority of Y434C mutant individual cell lines expressed a low level of mature form (145 kDa) TrkB protein (Fig. 1*D*). Some Y434C cell lines (#s 10, 12, 22, and 24) exhibited high levels of expression of proteins migrating at sizes ranging from approximately 40 to 120 kDa (Fig. 1*D*). Other lines (*e.g*., including #23, Fig. 1*D*) exhibited low levels of TrkB proteins of any size. Notably, a small subset (2/25) of Y434C mutant individual cell lines exhibited some level of mature form (145 kDa) (#6, Fig. 1*D*; #15, Fig. 2*C*).

The migration patterns detected in lysates of the pooled Y434C cell line (Fig. 1*B*) were not commonly observed in the individual cell lines (Fig. 1*D*). One explanation is that the migration pattern may evolve during passages of the cells isolated from the initial culture. To test these possibilities, 12 subclones were subsequently selected from a single individual cell line (TrkB Y434C #24, Fig. 1*D*). Following at least five passages, lysates of each of the 12 subclones were prepared, and the migration patterns of Y434C proteins on SDS-PAGE were analyzed by immunoblotting with the N-terminal TrkB antibody (Fig. 1*E*, *top*). While two major immunoreactivities at approximately 90 and 60 kDa were detected in the parent cell line (TrkB Y434C #24, Fig. 1*D*), the patterns of the 12 subclones were strikingly heterogeneous (Fig. 1*E*, *top*). Some exhibited a 60 kDa band (#s 9 and 10), others predominantly 30 KDa (#s 3, 5, 11, and 12), and others little detectable immunoreactivity (#s 4, 6, and 8). The striking differences in the migration pattern of TrkB Y434C in the parent cell line (#24, Fig. 1*D*) compared with its subclones (Fig. 1*E*, *top*) reveal a dynamic evolution during multiple passages. This contrasts sharply with WT TrkB stable cell lines, including pooled and individual cell lines, which exhibit the same migration pattern (*e.g*., Fig. 1, *B* and *E*) despite multiple passages (*e.g*., compare WT TrkB in Fig. 1*D* *versus* 1E).

Also unexpected was the detection of TrkB Y434C by antibodies targeting the N but not the C terminus of the protein. In contrast to robust immunoreactivities detected with antibodies targeting the N terminus of TrkB (Fig. 1, *B*, *D* and *E*, *top*), the majority of 25 Y434C individual cell lines exhibited no detectable immunoreactivity when probed with an antibody targeting the FLAG epitope on the C terminus of TrkB (Fig. 1, *D* and *E*, *bottom*). Immunoreactivity was detected with the FLAG antibody in lysates from WT and cell line #s 6 and 22 (Fig. 1*D*, *bottom*) but not in other cell lines expressing substantial levels of mutant TrkB with sizes ranging from 40 to 90 kDa detected with the N-terminal antibody (Fig. 1*D*, *top*, line #s 10, 12, and 24). Similar results were obtained with an additional antibody targeting the C-terminal domain (amino acids 783–796) of TrkB (Fig. 1*E*, *middle panel*).

One explanation for the absence of detectable immunoreactivity by C-terminal TrkB antibodies was that the DNA sequence of the cDNA encoding the C-terminal fragment of TrkB was deleted during cell clone selection and/or cell culture. To address this issue, we used PCR to amplify TrkB cDNAs corresponding to the intracellular domain from the genomic DNA of Y434C stable cell line #24 (Fig. 1*F*, *top schematic*). Three sets of primers were able to amplify the expected sizes of PCR products from both TrkB plasmid DNA and genomic DNA of Y434C stable cells (Fig. 1*F*, *bottom panel*). These results demonstrate that TrkB cDNA remained intact in HEK cells stably expressing Y434C.

To assess the consequences of the Y434C mutation on TrkB receptor function, subsequent experiments examined the responsiveness to the addition of the canonical neurotrophin ligand, namely BDNF. Western blot analyses of lysates of the pooled WT cell line revealed that addition of BDNF induced striking time-dependent increases of phosphorylation of residues of both TrkB and Erk1/2, evidence of BDNF-mediated activation of TrkB signaling in heterologous cells (Fig. 2, *A* and *B*). By contrast, addition of BDNF to the pooled Y434C cells induced virtually no increase in phosphorylation of residues of either TrkB or Erk1/2 (Fig. 2, *A* and *B*). These results were confirmed and extended in studies of individual cell lines of WT and Y434C (Fig. 2*C*). Similar to the pooled stable cell line, addition of BDNF to a clonal cell line expressing WT induced time-dependent increase of phosphorylation of TrkB, paralleled by increased phosphorylation of both Erk and phospholipase Cγ1 (Fig. 2*C*). Addition of BDNF to a Y434C cell line that expressed mature (145 kDa) TrkB protein (#15, Fig. 2*C*) induced a minimal increase of phosphorylated TrkB, phosphorylated phospholipase Cγ1, and phosphorylated Erk1/2 (Fig. 2*C*). Interestingly, the time course of the BDNF response in cell line #15 differed from that of the WT TrkB cell line in that the increases had largely reverted to basal level by 60 min in the mutant (Fig. 2*C*). Together, analyses of the pooled and individual cell lines reveal that expression of Y434C impairs TrkB signaling in the heterologous cells.

### What is the biochemical mechanism underlying the defective TrkB properties?

**Figure 3:**
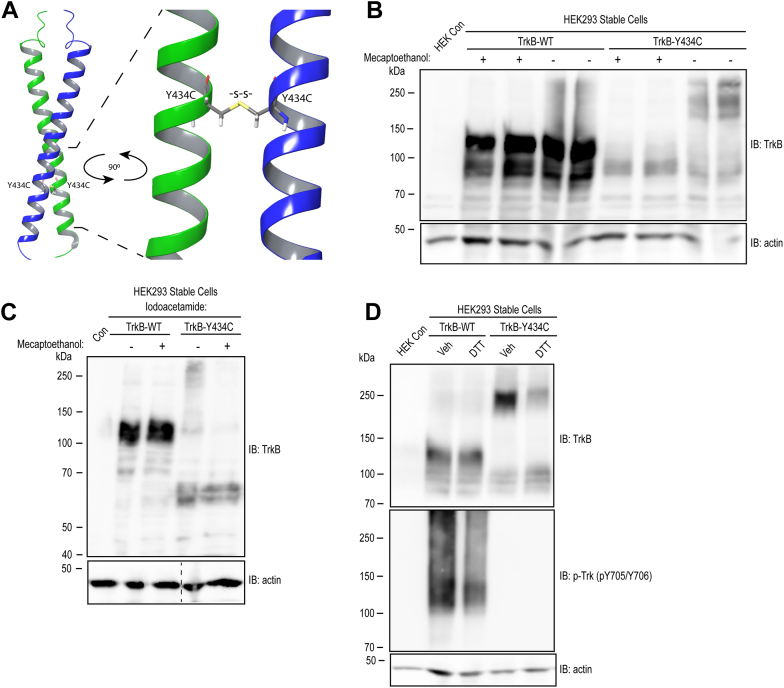
**Evidence of disulfide bond–mediated dimerization of TrkB Y434C mutant protein.***A*, *in silico* modeling of the Y434C mutant protein dimer using AlphaFold 3 software. Shown is a configuration of a segment of the transmembrane domain of two Y434C mutant proteins. A disulfide bond (highlighted in *stick*) formed between cysteines at amino acid 434. *B* and *C*, cell lysates of TrkB WT and Y434C *pooled* cell lines were prepared in the absence (*B*) and presence (*C*) of iodoacetamide and then mixed with either vehicle or a reducing agent β-mercaptoethanol (β-ME), prior to loading onto 8% SDS-PAGE. The migrating pattern of WT proteins remained unchanged in the absence and presence of β-ME. By contrast, in the absence of β-ME treatment, bands migrating at approximately 180 to 250 kDa were detected in the Y434C mutant protein; following treatment with β-ME, these bands shifted to approximately 90 kDa. *D*, both WT and Y434C cells themselves were briefly (15 min) treated with vehicle or a reducing agent (DTT, 100 μM) and then lysed with radioimmunoprecipitation buffer and resolved on SDS-PAGE in the absence of β-mercaptoethanol. Treatment of cells with DTT induced a 250 to 90 kDa band shift for the Y434C mutant protein but did not modify the migration of the WT protein. β-actin image in *C* was developed from a separate blot. The *dashed line* denotes the position at which irrelevant lanes were removed from the images.

Conversion of tyrosine in the WT to cysteine in the mutant Y434C raised the possibility that mutant-specific disulfide bonds form between the Y434C proteins. To assess this possibility, we conducted *in silico* modeling of protein complex structures of two molecules of the transmembrane domain of either WT or Y434C. The algorithm generated a structure of two helical forms of WT molecules similar to the structure experimentally defined by Kot *et al.* (12). By contrast, the algorithm predicted formation of an intermolecular disulfide bond when cysteine was substituted for tyrosine (Y434C) (Fig. 3*A*). In addition to these studies restricted to the transmembrane domains, *in silico* modeling was also deployed to examine the full lengths of WT and Y434C TrkB sequences. Consistent with prior publications (13), the model predicted six intramolecular disulfide bonds within the ectodomain, but no intermolecular disulfide bonds formed between WT proteins. In contrast, the model predicted an intermolecular disulfide bond between cysteine 434 of the Y434C protein in addition to the intramolecular disulfide bonds in the ectodomain.

These *in silico* findings led to biochemical experiments to assess the possibility of intermolecular disulfide bonds of Y434C TrkB. We first analyzed the SDS-PAGE migration pattern of TrkB prepared from lysates of the pooled WT and Y434C cell lines that were mixed with β-mercaptoethanol, a reducing agent or vehicle prior to loading on SDS-PAGE gels (Fig. 3*B*, *left*). Migration patterns of WT TrkB protein on SDS-PAGE were similar in the presence and absence of a reducing agent (Fig. 3*B*). Y434C mutant protein from the pooled cell line migrated at predominantly 90 kDa in the presence of a reducing agent (Figs. 1*B*, 3*B*). In contrast, in the absence of a reducing agent, bands migrating at approximately 180 to 270 kDa were evident in Y434C lysates in addition to a low level of 90 kDa (Fig. 3*B*), raising the possibility of intermolecular disulfide bonds promoting the formation of multimers of the Y434C mutant protein. Consistent with this idea, incubating Y434C cell lysates with a reducing agent, prior to electrophoresis on SDS-PAGE, resulted in a band shift from the size of 180 kDa or higher to 90 kDa (Fig. 3*B*). Analysis of additional Y434C individual cell lines revealed similar results, suggesting formation of multimers of Y434C mutant protein (Fig. 3*C*). To exclude the possibility of disulfide bonds simply forming during the experimental procedure, additional experiments included iodoacetamide in the lysis buffer, thereby enabling covalent bonds of iodoacetamide with the free thiol of cysteines concurrent with cell lysate preparation. Despite the presence of iodoacetamide in the lysis buffer, multiple bands of Y434C proteins migrating approximately at 60, 120, and 240 kDa were detected in cell lysates of Y434C cell line #24 (Fig. 3*C*). Incubation of this lysate with β-mercaptoethanol induced a shift of high molecular weight bands to 60 kDa (Fig. 3*C*).

We further examined the possibility of disulfide bond formation of Y434C TrkB in living cells, prior to preparation of cell lysates. Toward that end, a reducing agent was added to cultured cells themselves, followed by SDS-PAGE in the absence of β-mercaptoethanol. Incubating Y434C cells briefly (15 min) with reducing agent DTT diminished the 180 kDa band and increased the bands migrating at 90 kDa (Fig. 3*D*, *top*). By contrast, DTT did not modify the migration of bands at approximately 145 kDa in lysates from WT cells. In sum, the presence of high molecular weight forms of Y434C but not WT protein, together with the sensitivity of these protein species to treatment with reducing agents, is consistent with the possibility of disulfide bonds forming between the Y434C but not TrkB proteins. Remarkably, despite the presence of disulfide bond–mediated formation of Y434C TrkB dimers/multimers, this did not induce activation of the TrkB receptor as evidenced by the absence of detectable phosphorylation of tyrosines (Y 705/Y706) in contrast to WT TrkB protein (Fig. 3*D*, *bottom*).

**Figure 4:**
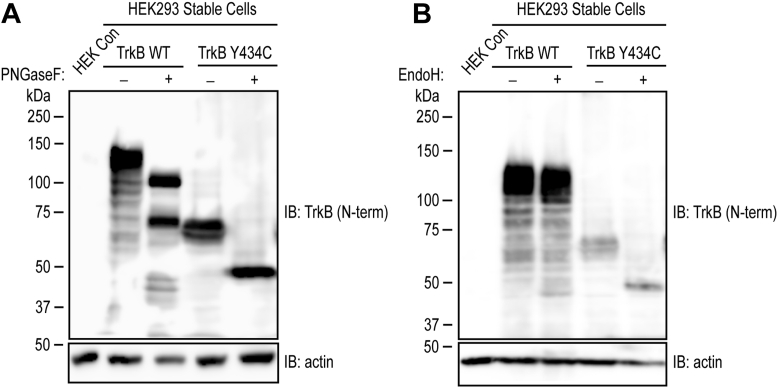
**Evidence of glycosylation of both TrkB WT and Y434C mutant proteins.** Cell lysates of TrkB WT and Y434C stable cell line (#24) were incubated with vehicle or a glycosidase (PNGase in *A*; EndoH in *B*) for 1 h and resolved on 8% SDS-PAGE. Incubation with PNGase induced a shift of WT protein from 145 to 90 kDa, whereas EndoH induced a minimal shift. In contrast, the addition of either PNGase or EndoH glycosidase induced a substantial shift of the Y434C mutant from 70 to 50 kDa.

One explanation for the limited activation of Y434C by BDNF in comparison to WT TrkB is an impairment of protein trafficking to the surface membrane of the cell. Glycosylation is a key step for membrane protein trafficking, raising the possibility that the Y434C mutant is abnormally glycosylated. To test this idea, we conducted an *in vitro* enzymatic analysis of WT and Y434C proteins using two glycosidases. We compared the effects of PNGase F, a glycosidase that removes all N-linked glycans, with those of Endo-H, a glycosidase that preferentially removes high mannose-rich glycans (14). With respect to WT, incubation of lysates with PNGase F induced a large shift of mature form Trk B from 145 to 90 kDa (Fig. 4*A*), whereas Endo-H induced only a small shift, consistent with previous reports (15, 16). With respect to Y434C, incubation of lysates with either PGNase F or Endo-H induced a shift of Y434C protein to a similar extent from approximately 60 kDa to 50 kDa. The fact that Endo-H induces a shift of Y434C similar to that of PGNase F implies that Y434C is enriched with high mannose-rich glycans, a post-translational modification of proteins localized predominantly to intracellular membranes, including endoplasmic reticulum and Golgi (17). The minimal effect of Endo-H on the WT TrkB protein migrating at 145 kDa is consistent with the relative paucity of mannose-rich glycans in proteins localized predominantly to the cell membrane.

## Discussion

Insight into the biochemical properties of the Y434C TrkB receptor is an important initial step in understanding how this mutation causes a devastating phenotype. This led us to establish and characterize multiple cell lines stably expressing either WT or Y434C TrkB. Three principal findings emerged. In comparison to WT, Y434C mutant cell lines expressed low levels of mature forms of (145 kDa) TrkB protein and increased levels of forms migrating at sizes ranging from 40 to 110 kDa under reducing conditions. High molecular weight forms consistent with multimers of Y434C were found by SDS-PAGE conducted under nonreducing conditions; addition of reducing agents reduced the migration patterns. Mutant cell lines exhibited striking impairments of BDNF-mediated activation of TrkB signaling. Y434C mutant protein is enriched with *N*-linked high mannose-rich glycans evidenced by a glycosidase assay. We conclude that the Y434C point mutation promotes formation of multimers of the mutant proteins through intermolecular disulfide bonds and thereby enhances their abnormal proteolytic digestion with at least two major biochemical consequences: (a) the virtual absence of the mature (145 kDa) form of TrkB and impaired BDNF-activated TrkB signaling and (b) the generation of a diversity of proteolytic fragments of TrkB containing N-terminal ectodomain. We suspect that brief overexpression of TrkB protein following transient transfection in heterologous cells confounded characterization of the biochemical properties of the Y434C mutant protein in earlier studies (10) and accounts for differences from the results reported here.

Our results provide a clue to the structural and functional consequences of substituting cysteine for Y434 of TrkB, a residue that lies within the transmembrane domain of this receptor tyrosine kinase. Substitution of cysteine in particular has deleterious consequences in that substitution of neither leucine nor alanine modified the transmembrane helical structure of TrkB or BDNF-mediated TrkB activation in heterologous cells (12). Indeed, no abnormalities were evident in a genetically modified mouse in which phenylalanine was substituted for Y434 (18). *In silico* modeling predicted the presence of intermolecular disulfide bonds between Y434C TrkB monomers (Fig. 3*A*). The presence of high molecular weight forms of Y434C TrkB in SDS-PAGE under nonreducing conditions, together with reductions of their size induced by reducing agents, supports this prediction. Neurotrophin-induced formation of homodimers promotes activation and downstream signaling of WT TrkB (2). Yet despite the likely presence of homodimers of Y434C proteins as well as additional multimers (Fig. 3*D*, *top*), these disulfide-mediated dimers did not evoke activation of TrkB (Fig. 3*D*, *bottom*). These findings suggest that mutation of this residue within the transmembrane domain modifies the structure of cytoplasmic domains that limit activation of signaling. In addition to limiting signaling, we suggest that structural abnormalities of the cytoplasmic domain of Y434C proteins create substrates for a diversity of proteases.

The presence of proteolytic degradation products of TrkB evident in Y434C cell lines supports this suggestion. That is, analyses of migration patterns of lysates of cell lines stably expressing Y434C TrkB revealed not only reductions of mature form (145 kDa) TrkB but also increases of lower molecular weight N-terminal fragments of TrkB. These fragments are not simply generated by truncation of 3′ TrkB cDNA *via* genomic integration during clone selection and/or genomic instability, in part because PCR experiments establish the integrity of the DNA sequence in Y434C cells. Rather, these fragments of TrkB are almost certainly because of proteolytic digestion of Y434C proteins. These fragments exhibit striking diversity in size, ranging from 40 to 110 kDa (Fig. 1*E*). The size of these fragments is dynamic in that it evolves over multiple passages from the same single cell clone and exhibits heterogeneity in the size of fragments among subclones of the same originating line (Fig. 1*E*). Notably, the vast majority of fragments were detected with an antibody targeting the N terminus, whereas few or no fragments were detected with antibodies targeting the C terminus. Whether this is due to the limited sensitivity of these antibodies, the absence of the antibody’s epitope in the fragment and/or the absence of the C-terminal fragments is unclear.

Work by others suggests that the lower molecular weight fragments of TrkB in the Y434C TrkB cell lines may themselves have toxic consequences. Proteolytic degradation of TrkB has been identified in animal models of Alzheimer’s disease and demonstrated directly in the neocortical tissue of humans with this disease (15, 16). Importantly, viral-mediated expression of an intracellular fragment of TrkB is sufficient to produce both structural and functional defects of cultured cortical neurons (15). Moreover, a synthetic short peptide that limits proteolytic degradation of TrkB reduces cognitive deficits in a mouse model of Alzheimer’s disease (15). Collectively, these findings support the proposal that proteolytic degradation products of TrkB may have deleterious consequences.

Might proteolytic degradation products of Y434C TrkB contribute to the phenotypic manifestations of humans carrying this mutation? A single copy of the Y434C mutation causes a remarkably stereotyped collection of disorders dominated by medically refractory epileptic seizures in the first year of life (8). The seizure disorder is accompanied by impaired cognitive and motor development, autistic behavior, and visual impairments. Neither loss nor gain-of-function mutations of BDNF or WT TrkB introduced into mice cause similar phenotypes (1, 3). While transgenic overexpression of BDNF or TrkB enhances susceptibility to epilepsy, neither is sufficient to produce other features of the human phenotype such as motor or visual impairments (19, 20). An alternative explanation for the devastating epileptic encephalopathy is that the Y434C mutation causes a gain of toxic function. That N-terminal fragments of Y434C TrkB are enriched with immature glycans (Fig. 4) suggests that the Y434C mutant protein is likely restricted to intracellular compartments because of impaired cell surface trafficking. If the toxic consequences of fragments of Y434C TrkB cause this devastating phenotype, identification of the protease (s) degrading Y434C TrkB and its cleavage site (s) will provide molecular targets for disease-modifying therapies.

## Experimental procedures

### Reagents and plasmids

Recombinant human BDNF was purchased from Millipore (GF301). Cultured Dulbecco's modified Eagle's medium (11995-065), fetal bovine serum (A5670701), and geneticin (11811-031) were purchased from Gibco. PNgase F was purchased from New England Biolabs (P0704). Additional reagents were obtained from Sigma, unless specified otherwise. The mammalian expression plasmid of mouse TrkB pDK6-TrkB (WT) was described previously, in which a FLAG epitope tag was appended to the C terminus of TrkB (21). The Y434C point mutation was incorporated into pDK6-TrkB using a PCR-based QuickChange site-directed mutagenesis method (Stratagene). Plasmid DNAs were purified with QIAGEN Plasmid Maxi kit (12163).

### Cell culture and transfection

HEK293T cells were obtained from American Type Culture Collection through Duke Cell Culture Facility and cultured in Dulbecco's modified Eagle's medium supplemented with 10% fetal bovine serum, including 1% penicillin and streptomycin, at 37 °C in the presence of 95% O_2_/5% CO_2_. For transfection experiments, cells were transfected with either empty vector, TrkB-WT, or TrkB-Y434C mutant plasmid DNA (1 μg) using the Lipofectamine method (Gibco).

### TrkB WT and Y434C stable cell lines

Twenty-four hours after transfection, cells were cultured in the presence of geneticin (1 mg/ml) for 2 weeks. Cells from 25 single clones were manually retrieved for further study, and the remaining cells, including the residuals of the 25 single clones, were pooled and cultured and referred to as “pooled cell lines” (Fig. 1*B*). Cells from each of the 25 single clones were grown in 12-well culture plates and defined as “individual cell lines” (Fig. 1*D*). Finally, subclones of one of the 25 individual cell lines (TrkB Y434C #24 Fig. 1*D*) were further selected and analyzed (Fig. 1*E*). “Pooled cell lines,” “individual cell lines,” and “subclones” of TrkB Y434C #24 were cultured in the presence of a low concentration of geneticin (0.4 mg/ml).

### Genomic DNA purification and PCR

Genomic DNA of HEK Y434C cell line #24 was purified using EZ-10 Spin Column Genomic DNA Minipreps Kit (Bio Basic). Fragments corresponding to the C terminus of the Y434C protein were amplified by PCR using primers highlighted in Figure 1*E*. PCR products were purified using QIAquick PCR Purification Kit (QIAGEN) and sequenced by Eton Bioscience.

### Immunoblotting

Cells were washed with PBS buffer (pH 7.4, Invitrogen) and lysed in modified radioimmunoprecipitation assay buffer (50 mM Tris–HCl, pH 7.4, 150 mM NaCl, 1% NP-40, and 0.25% sodium deoxycholate) and proteinase inhibitor cocktail (Roche). In some experiments, 10 mM iodoacetamide was added into the radioimmunoprecipitation assay before cell lysis. After centrifuging at 16,000*g* for 10 min, the supernatant was collected as the cell lysate. Protein concentration was determined by the BCA protein assay (Pierce). Cell lysates (10–20 μg) were suspended in Laemmli buffer with a reducing agent like β-mercaptoethanol and resolved by 8% SDS-PAGE. For nondenaturing electrophoresis (SDS-PAGE), β-mercaptoethanol was excluded from the samples. Blots were incubated overnight with primary antibodies and subsequently with secondary antibodies (1:5000 dilution) for 1 h at room temperature. Western blots were developed with Molecular Imager (Amersham). The following TrkB antibodies were employed in this study: N-terminal TrkB, mouse monoclonal (immunogen, amino acids 156–322), 1:500 dilution, catalog #610101, BD Transduction Laboratories; N-terminal TrkB, rabbit monoclonal (80E3) (immunogen, a synthetic short peptide surrounding amino acid 50), 1:1000 dilution, catalog #4603, Cell Signaling; C-terminal TrkB, rabbit monoclonal targeting FLAG epitope, 1:500 dilution, catalog #14793, Cell signaling; C-terminal TrkB, rabbit polyclonal (immunogen, a peptide corresponding to 14 amino acids between 783 and 796 of TrkB), 1:250 dilution, catalog #sc-11, Santa Cruz. Other antibodies and dilution used in this study are as follows: p-Trk (pY515), p-Trk (pY705/706), p-Akt, p-Erk (1:1000 dilution, Cell Signaling), and β-actin (1:10,000 dilution; Sigma). The specificity of each TrkB antibody was verified by including HEK293 plain cell lysate as a negative control and WT TrkB cell lysate as a positive control in each experiment. The immunoblots were developed using enhanced chemiluminescence primary Western blotting detection reagents (Cytiva) on Amersham Imager 600. Verification of equivalent loading and transfer was assessed with immunoblotting for β-actin. β-actin blots were developed by reblotting original gels or separate gels. Shown are representative results of immunoblotting from at least three independent experiments. The density of immunoreactivities was quantified by ImageJ software (National Institutes of Health).

### *In vitro* protein deglycosylation

An *in vitro* deglycosylation assay was performed as recommended by the manufacturer (New England BioLabs). In brief, 50 to 100 μg cell lysates were denatured by heating at 100˚C for 10 min. Denatured proteins were digested with PNGase F (catalog #P0704) or Endo H (catalog #P0702) (1000 units) at 37 °C for 60 min. The extent of deglycosylation was assessed by mobility shifts on SDS-PAGE gels following a Western blot analysis.

### *In silico* protein structure prediction

The structures of the full-length or transmembrane domain (amino acids 423–466) of human WT (unique ID: Q16620) and Y434C TrkB protein were analyzed using the software of AlphaFold 3 (22). Unsupervised modeling of two molecules of either the WT or the Y434C protein was conducted without constraints. Details of structures were also visualized and analyzed by Maestro 14.5 and PyMol 3.1 software (Schrödinger, LLC).

### Statistical analysis

Data are presented as mean with standard error of the mean. The method of statistical analysis is indicated in the figure legends. Statistical tests were performed using GraphPad Prism (version 10.6.0; Dotmatics).

## Data availability

Data presented in this article will be available upon reasonable request.

## Conflict of interest

The authors declare that they have no conflicts of interest with the contents of this article.
